# Supplementary material for: Effluent Molecular Analysis Guides Liver Graft Allocation to Clinical Hypothermic Oxygenated Machine Perfusion
Source: Biomedicines. 2021 Oct 11;9(10):1444. doi: 10.3390/biomedicines9101444 (PMC8533371; doi:10.3390/biomedicines9101444)
Supplement: Supplementary file 1 [file biomedicines-09-01444-s001.zip › biomedicines-1386670-supplementary.pdf]

Supplementary material

Table of Contents

Supplementary methods **.......... 2**

Supplementary Figure S1. Study design. 2

Supplementary Figure S2. Calibration curves used to measure concentration of FMN in unknown biological samples. 3

Supplementary Table S1. Low standards, high standards, and sensitivity of the tests used in the present study to assess analyte concentration in effluent fluids. 4

Supplementary results **.......... 5**

Supplementary Figure S3. Color map on correlations across the parameters evaluated in the present study. 5

Supplementary Table S2. Differences in the molecular profile of effluent fluids of livers from the "EAD-no" and the "EAD-yes" groups. 6

Supplementary methods

Supplementary Figure S1. Study design.

Schematic representation of effluent fluid collection at the end of the back-table. Liver graft was subjected to cold flush through the portal vein and, after complete cava vein clamping, the effluent was collected using an cannula inserted in the posterior aspect of the retro-hepatic cava vein. A) Timeline of organ donation process and liver preservation; effluent fluid collection is indicated. B) Representative picture showing flushing through the portal vein and the vena cava clamping

A

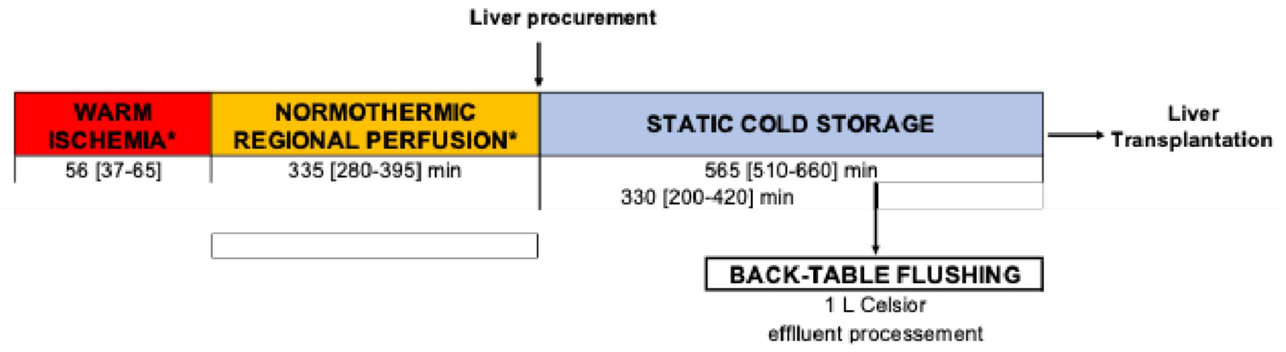

B

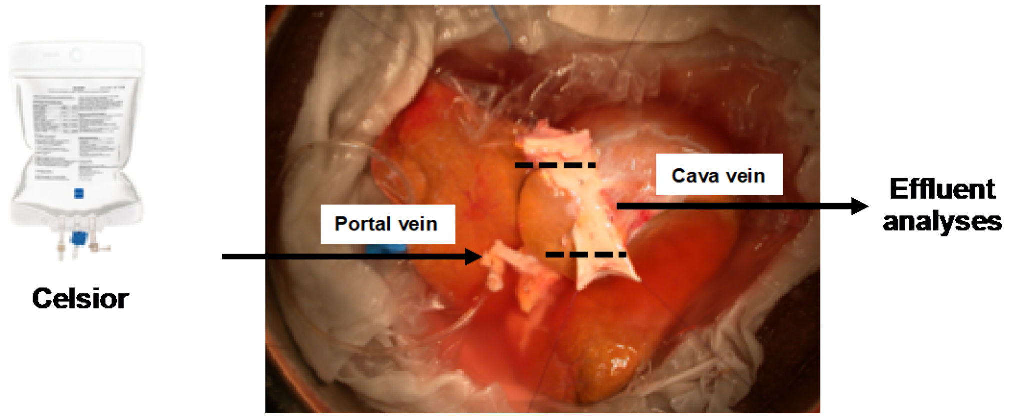

Supplementary Figure S2. Calibration curves used to measure concentration of FMN in unknown biological samples.

One hundred fifty microliters of standard samples were dispensed in triplicate in black plates. Excitation wavelength was set at 460/40 nm, while fluorescence emission was revealed with 100% gain at 528/20 nm.

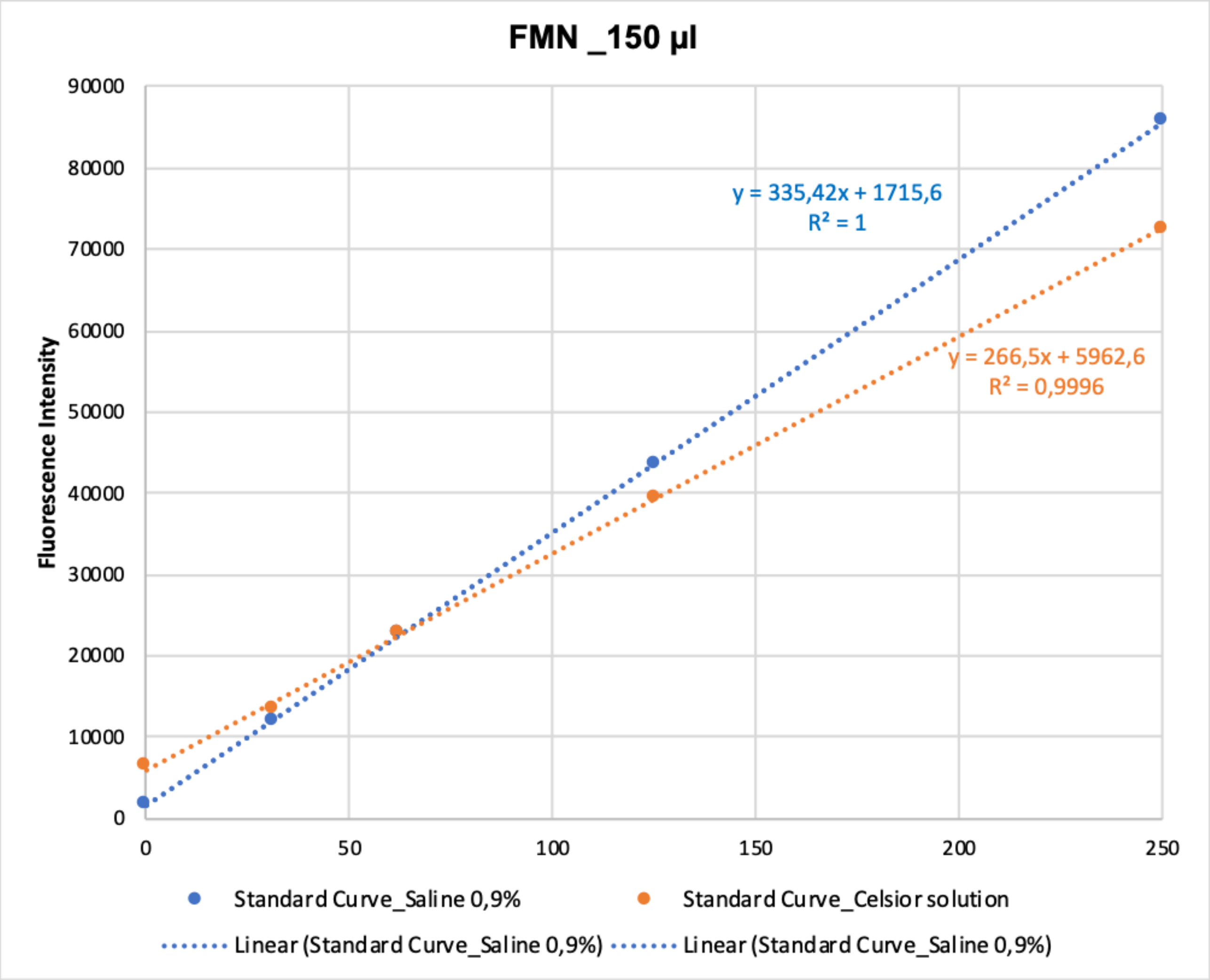

Supplementary Table S1. Low standards, high standards, and sensitivity of the tests used in the present study to assess analyte concentration in effluent fluids.

Analyte	Method	Assay range	Sensitivity	Units	Company
Angiopoietin-like Protein-3/ANGPTL3	Human Magnetic Luminex® Screening Assay*	267-65000	23	pg/ml	R&D Systems
Angiopoietin-like Protein-4/ANGPTL4	Human Magnetic Luminex® Screening Assay*	1850-450000	86	pg/ml	R&D Systems
Tumor Necrosis Factor/TNF-alpha	Human Magnetic Luminex® Screening Assay*	8,23-2000	1,2	pg/ml	R&D Systems
Coagulation Factor XIV/Protein C	Human Magnetic Luminex® Screening Assay*	206-50000	23,1	pg/ml	R&D Systems
Hepatocyte Growth Factor/HGF	Human Magnetic Luminex® Screening Assay*	16,5-4000	1	pg/ml	R&D Systems
C-X-C Motif Chemokine Ligand 9/CXCL9/MIG	Human Magnetic Luminex® Screening Assay*	617-150000	23,8	pg/ml	R&D Systems
C-C Motif Ligand 2/CCL2/MCP-1	Human Magnetic Luminex® Screening Assay*	30,9-7500	9,9	pg/ml	R&D Systems
Interleukin 1 beta/IL-1 beta/IL-1F2	Human Magnetic Luminex® Screening Assay*	17,7-4300	0,8	pg/ml	R&D Systems
Interleukin 10/IL-10	Human Magnetic Luminex® Screening Assay*	4,12-1000	1,6	pg/ml	R&D Systems
Interleukin 6/IL-6	Human Magnetic Luminex® Screening Assay*	4,53-1100	1,7	pg/ml	R&D Systems
Interleukin 8/IL-8/CXCL8	Human Magnetic Luminex® Screening Assay*	4,12-1000	1,8	pg/ml	R&D Systems
Tumor Necrosis Factor/TNF-alpha	Enzyme-Linked Immunosorbent Assay (ELISA)	15,6-1000	5,5	pg/ml	R&D Systems
Serpin E1/PAI-1	Enzyme-Linked Immunosorbent Assay (ELISA)	0,313-20	0,059	ng/ml	R&D Systems
Galectin-9	Enzyme-Linked Immunosorbent Assay (ELISA)	0,156-10	0,08	ng/ml	R&D Systems
Glypican 3	Enzyme-Linked Immunosorbent Assay (ELISA)	78-5000	9,18	pg/ml	R&D Systems
Pentraxin3/TSG-14	Enzyme-Linked Immunosorbent Assay (ELISA)	0,313-20	0,014	ng/ml	R&D Systems
Hyaluronan	Enzyme-Linked Immunosorbent Assay (ELISA)	0,6-40	0,068	ng/ml	R&D Systems
Syndecan-1/CD138	Enzyme-Linked Immunosorbent Assay (ELISA)	8-256	4,94	ng/ml	Diaclone

*Human Premixed Multi-Analyte Kit

Supplementary results

Supplementary Figure S3. Color map on correlations across the parameters evaluated in the present study.

Color scale visually represents values of Pearson correlation coefficients.

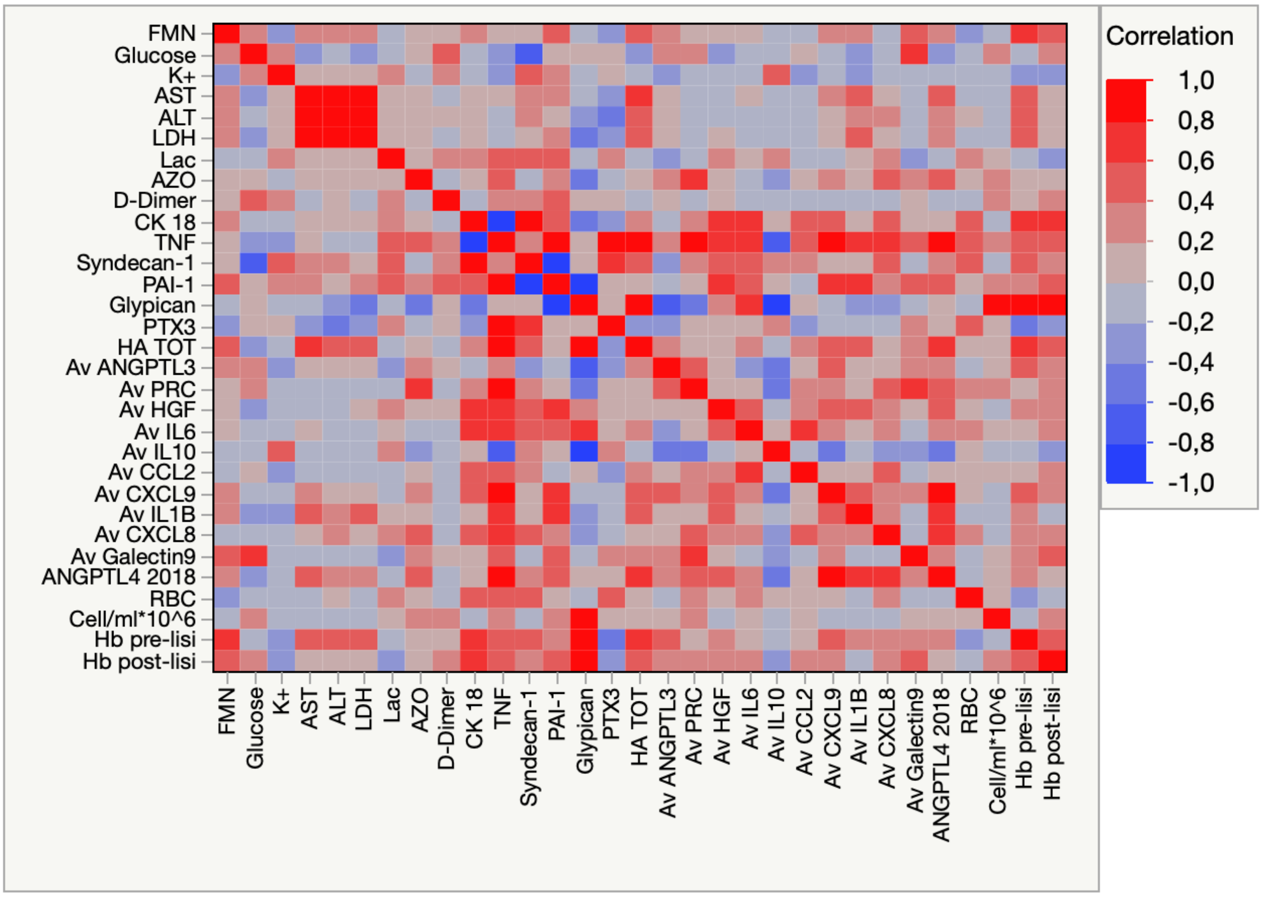

Supplementary Table S2. Differences in the molecular profile of effluent fluids of livers from the "EAD-no" and the "EAD-yes" groups.

Results are presented as Median [25-75 percentiles]; p values were calculated using the non-parametric Wilcoxon test.

Pattern	Variable	EAD-no	EAD-yes	p value
**Inflammation**				
	CCL2/MCP-1, pg/g	136.100 [69.200-238.700]	249.500 [62.350-379.925]	0.6501
	CXCL8/IL-8, pg/g	11.450 [6.000-23.750]	9.800 [5.000-23.900]	0.8528
	CXCL9/MIG, pg/g	79.30 [54.525-115.225]	171.000 [25.500-214.975]	0.4013
	IL-1b, pg/g	4.800 [2.750-8.662]	10.000 [4.700-15.250]	0.2316
	IL-6, pg/g	34.225 [16.100-58.950]	42.200 [13.750-64.275]	0.9179
	PTX3, ng/g	0.0950 [0.0395-0.189]	0.0200 [0.0163-0.0957]	-
	TNF-a, pg/g	2.150 [0.400-3.400]	1.950 [1.100-3.850]	0.8260
	IL-10, pg/g	3.250 [0.750-17.700]	2.100 [0.650-8.025]	0.8851
**Resolution, Repair, Regeneration**				
	ANGPTL3, pg/g	592.700 [321.250-1272.100]	1238.400 [352.325-1964.475]	0.4351
	ANGPTL4, ng/g	1.000 [0.500-57.200]	40.000 [3.575-108.750]	0.4694
	Galectin-9, ng/g	3.325 [1.200-6.000]	5.600 [1.700-6.950]	0.6159
	HGF, pg/g	494.600 [367.400-848.025]	403.300 [222.700-439.300]	0.2305
**Coagulation**				
	Protein C/Factor IVX, pg/g	130.125 [93.000-306.400]	160.100 [63.100-218.650]	0.7887
	D-dimer, mg/g	1.478 [0.757-2.990]	1.024 [0.688-2.429]	0.3041
	PAI-1, ng/g	0.136 [0.0355-0.171]	0.138 [0.0450-0.231]	0.2986
**Hepatocyte injury**				
	CK18, U/g	0.214 [0.151-0.813]	1.070 [0.346-3.475]	0.4413
	AST, IU/g	0.288 [0.207-0.450]	0.708 [0.323-1.415]	**0.0341**
	ALT, IU/g	0.222 [0.168-0.425]	0.553 [0.349-0.976]	**0.0478**
	LDH, IU/g	0.527 [0.347-0.829]	1.320 [0.656-2.496]	**0.0478**
**Liver/hepatocyte metabolism**				
	BUN, mmol/g	0.00200 [0.00200-0.00500]	0.00200 [0.00100-0.00275]	0.1689
	Lac, mmol/g	0.00300 [0.00200-0.00300]	0.00200 [0.00200-0.00200]	0.2766
	FMN, ng/g	33.500 [20.600-50.325]	37.600 [33.100-52.000]	0.2464
**Glycocalyx**				
	Glypican, pg/g	38.688 [24.973-66.222]	NA	-
	HA, ng/g	12.350 [6.900-24.300]	34.150 [12.600-108.000]	0.1171
	Syndecan-1, pg/g	420.200 [283.200-470.600]	504.500 [412.050-636.400]	0.4014
**Other**				
	K+, mmol/g	0.0145 [0.0120-0.0160]	0.0120 [0.0103-0.0120]	**0.0156**

Free hemoglobin, mcg/g	9.900 [1.100-20.500]	21.100 [13.600-23.825]	0.0900
WBC, cells/g*10^6	0.600 [0.400-0.975]	0.400 [0.200-0.700]	0.2973
RBC, cells/g*10^3	362.000 [165.900-568.200]	151.900 [94.700-269.500]	0.2023
